# Correlation between Official and Common Field-Based Fitness Tests in Elite Soccer Referees

**DOI:** 10.3390/jfmk6030059

**Published:** 2021-07-01

**Authors:** Veronica Romano, Manuel Tuzi, Ada Di Gregorio, Anna Maria Sacco, Immacolata Belviso, Felice Sirico, Stefano Palermi, Daria Nurzynska, Franca Di Meglio, Clotilde Castaldo, Angelo Pizzi, Stefania Montagnani

**Affiliations:** 1 Department of Public Health, University of Naples “Federico II”, 80131 Naples, NA, Italy; tuzimanuel@gmail.com (M.T.); adadigregorio.nutrizione@gmail.com (A.D.G.); annamaria.sacco@unina.it (A.M.S.); immacolata.belviso@unina.it (I.B.); felice.sirico2@unina.it (F.S.); stefano.palermi@unina.it (S.P.); franca.dimeglio@unina.it (F.D.M.); clotilde.castaldo@unina.it (C.C.); montagna@unina.it (S.M.); 2 Department of Medicine, Surgery and Dentistry “Scuola Medica Salernitana”, University of Salerno, 84081 Baronissi, SA, Italy; dnurzynska@unisa.it; 3 Italian Association of Referees (AIA, Associazione Italiana Arbitri)–FIFA Referees Medical Committee, 00185 Rome, RM, Italy; angelopizzi@alice.it

**Keywords:** soccer, referee, fitness, Illinois agility test, hand-grip strength

## Abstract

Official tests are used to assess the fitness status of soccer referees, and their results correlate with match performance. However, FIFA-approved tests expose the referees to high physical demands and are difficult to implement during the sportive year. The aim of our study was to evaluate the correlation between the 6 × 40-m sprint and Yo-Yo Intermittent Recovery Level 1 (IR1) official tests and other field-based tests that require no or little equipment, are not time-consuming, and impose low physical demands. All tests were performed by male referees from the Regional Section of the Italian Referee Association (*n* = 30). We observed a strong correlation between 6 × 40-m sprint and Illinois agility tests (r = 0.63, *p* = 0.001) and a moderate correlation between Yo-Yo IR1 and hand-grip strength in the dominant (r = 0.45, *p* = 0.014) and non-dominant hand (r = 0.41, *p* = 0.031). Interestingly, only a moderate correlation (r = −0.42, *p* = 0.025) was observed between the FIFA official tests, 6 × 40-m sprint and Yo-Yo IR1. These results suggest that Illinois agility and hand-grip tests could represent simple and low-physical-impact tools for repeated assessment and monitoring of referee fitness throughout the sportive season.

## 1. Introduction

The referee is in overall control of the soccer game. In addition to the considerable psychological and cognitive demands placed on referees during games [1], an extensive load is imposed on their cardiovascular and musculoskeletal systems. However, it is the training, performance, and injury prevention of soccer players that have been extensively studied over the last few decades, with only limited scientific studies dedicated to fitness monitoring and injury prevention in referees. Importantly, referees are usually not full-time professionals, they are on average 15–20 years older than players, and they cannot normally be substituted. During the game, they perform a mix of walking and running activity of low, medium, and high intensity [2,3]. Hence, the physical fitness of elite soccer referees is of fundamental importance for effective officiating.

National and international soccer referees’ associations routinely assess the performance of elite-level officials. Several field-based tests have been used for years to this aim. Following the reports on the poor validity of the 2 × 50-m sprint, 2 × 200-m sprint, and 12-min Cooper tests [4,5] in measuring the referees’ match-related physical capacity, the Fédération Internationale de Football Association (FIFA) introduced a 6 × 40-m sprint test and a high-intensity 150-m interval test as official fitness tests for men and women referees [6]. The intensity of the latter was subsequently reduced in the latest guidelines, which now recommend 40 × 75-m run/25-m walk intervals [7].

While the validity of the 6 × 40-m sprint test and its correlation with referees’ match performance were confirmed [8], only weak evidence exists for the high-intensity 150-m interval test [9], with comparison studies suggesting the advantage of the Yo-Yo Intermittent Recovery Level 1 (Yo-Yo IR1) test [10]. Subsequently, the validity of the latter test in determining the maximal activation of the aerobic system through intermittent exercise was observed [8]. As a result, the Yo-Yo IR1 can now be used in addition to the official tests as a method of assessing the aerobic fitness of referees, according to FIFA [6].

The fitness assessment is carried out at the beginning of the sportive year, and it defines the eligibility of referees for participation at the international, national, and regional levels. It is only rarely performed during the season. For one thing, the tight schedule can make it difficult for the active referees to participate in official assessments; indeed, even the training sessions between matches are often unsupervised and scheduled at the discretion of the participant. For another, the recommended tests are strenuous and physically demanding. In particular, the 6 × 40-m sprint test requires a maximal anaerobic muscular activation and the Yo-Yo IR1 test imposes muscular exhaustion. For these reasons, referees’ physical fitness for participation cannot be easily assessed during the training periods or intervals between the officiated soccer matches. Hence, simpler, less time-consuming, and less physically demanding tests could be potentially more feasible and useful for fitness screening during the sportive season.

The aim of this study was to evaluate the correlation between the results of the official fitness tests performed by AIA referees, namely 6 × 40-m sprint and Yo-Yo IR1, and other common tests aimed at the evaluation of several domains of physical fitness, such as: the hand-grip strength (HGS) test, which evaluates explosive strength in the upper limbs; the sit-and-reach (SaR) test, which assesses flexibility; the Illinois agility (IA) test, which assesses agility; and the standing long jump (SLJ) and standing quintuple jump (SQJ) tests, which both evaluate explosive strength in the lower limbs. Evidence of a valid correlation between these tests could allow the introduction of a fitness evaluation protocol that is easy to perform, consumes little time, and has low impact on subsequent physical activity. Such a protocol, then, could be used to evaluate referees during the sportive season to monitor their fitness level and guarantee the best possible performance and injury prevention during officiated matches.

## 2. Materials and Methods

### 2.1. Participants

The participants were enrolled in the study on a voluntary basis and represented a convenience sample of the Regional Section (Lazio) of the Italian Referee Association (Associazione Italiana di Arbitri, or AIA). All participants were male referees in the regional category of officiating, who participate in two to three training sessions per week and officiate one soccer match per week from September to May. Referees with active painful musculoskeletal complaints or with a history of a painful condition or surgery within the previous 6 months were excluded.

The study was approved by the Department Review Board for ethical concerns. All participants received an exhaustive explanation of the study protocol and objective. Each participant provided written informed consent prior to participation in the study and acknowledged that they cannot be identified via this paper because all data were made anonymous.

### 2.2. Study Protocol

**Figure 1:**
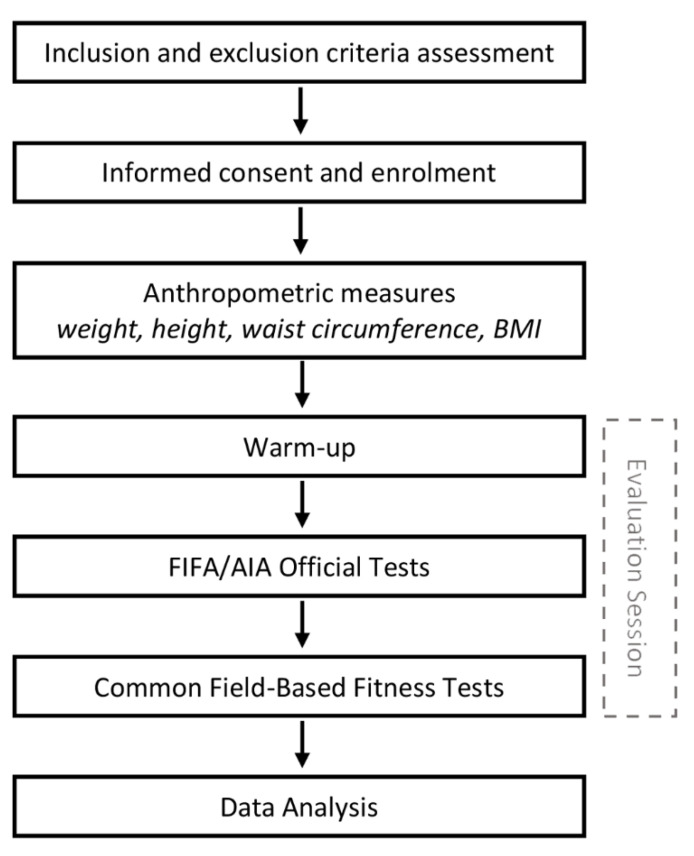
Study protocol flowchart.

The study protocol is graphically represented in Figure 1.

The study took place in September 2020. The participants were instructed to avoid vigorous physical activity during the previous 48 h and not to drink any caffeinated beverages during the previous 24 h before the evaluation session. A medical specialist in sport medicine obtained the medical history of each participant. Another doctor collected anthropometric data, including sex, age, dominant side, height, weight, and waist circumference. The body mass index was calculated in the standard way in kilograms per square meter. Each fitness evaluation session was performed on an artificial soccer field in one day and divided into three stages. The participants were familiarized with the testing procedures and verbally encouraged during test performance.

All activities were conducted according to the FIFA guidelines [6]. The participants were not allowed to wear spiked track shoes. The first stage consisted of a 20-min warm-up. A professional fitness coach, together with a sports medicine doctor, supervised these activities. The second stage comprised FIFA/AIA fitness tests. Each participant performed the 6 × 40-m sprint test, followed by the Yo-Yo IR1 test, with an 8-min interval between the two. The time of the fastest sprint during the first test, recorded in seconds, and the maximum distance covered during the second test, in meters, were used in the analysis. After completion of the official tests, the participants were allowed 15 min of rest. During this time, they could drink water freely, but no food or other beverages were allowed. The subsequent third stage of evaluation comprised the following field-based fitness tests: HGS, SLJ, SQJ, SaR, and IA test.

The HGS was measured with a digital dynamometer (Dynex, MD systems, Inc., Westerville, OH, USA) in the dominant and non-dominant arm, while the subject was standing with their shoulders in neutral position and their elbow flexed at 90 degrees [11]. Three measurements for each arm were made, with a recovery period of 30 s between repetitions. The best result obtained for each arm, expressed in kilograms, was considered for analysis.

The SLJ was performed with a two-foot take-off and landing, with swinging of the arms and bending of the knees allowed [12]. The distance was measured from the baseline to the point where the back of the heel nearest to the take-off line landed on the ground. The participants repeated the jump three times, and the longest distance was used for subsequent analysis. The SQJ required five consecutive two-foot jumps from a standing start. At each landing phase, the feet were aligned before the subsequent jump, based on the foot nearest to the baseline. After the last jump, the total distance from the start line was recorded in centimeters.

For the SaR test, the participant sat on the ground with knees fully extended and legs together. The soles of the feet were placed against a box, with the 23-cm point at the level of the contact. With arms extended and one hand placed on top of the other, the participant reached forward as far as possible without flexing their knees or moving their feet [13]. The final position of the hands, reached at the fourth trial, was recorded in centimeters from a measuring scale placed on top of the box.

The IA test was performed using the procedures outlined by Negra et al. [14]. The corners of a 10 m × 5 m field were marked with cones. Another four cones were placed down the center of the rectangle, 3.3 m apart. The participant remained prone on the ground with their hands at the shoulder level and their chin touching the starting line. On a verbal command, the referee got up and ran along the previously indicated course, turning between the cones. The completion time, expressed in seconds, was used for subsequent analysis.

### 2.3. Statistical Analysis

The results of the FIFA/AIA official tests were correlated with those of other field-based fitness tests using the Pearson correlation coefficient. All statistical analyses were performed with STATA software (StataCorp. v.12, College Station, TX, USA) by a researcher who was not involved in the data collection. The anthropometric measurements and the results of the fitness tests were considered as continuous data. Normality of the data distribution was confirmed using the Shapiro–Wilk test, and the results were reported as mean ± standard deviation. The level of statistical significance was set at *p* < 0.05. The datasets generated and analyzed during the current study are available from the corresponding author on reasonable request.

The null hypothesis was that no correlation exists between FIFA/AIA official tests and common field-based fitness tests, corresponding to a Pearson correlation coefficient = 0. The alternative 2-tailed hypothesis was that a large correlation was present between variables, corresponding to a Pearson correlation coefficient of at least 0.5. Admitting a type I error of 5% and a power of 80%, the minimum sample size, calculated according to Hulley et al. [15], was 29 subjects. The correlation was considered absent at r < 0.1. Higher values of the correlation coefficient were graded according to the following scale: weak, when r = 0.1–0.29; moderate, when r = 0.3–0.49; strong, when r = 0.5–0.69; very strong, when r = 0.7–0.9; and nearly perfect, when r > 0.9.

## 3. Results

Thirty-five referees volunteered to participate and were assessed for enrollment criteria. Five were excluded due to recent musculoskeletal injuries (three referees suffered an ankle sprain in the previous 2 months, one referee underwent a meniscal repair 4 months earlier, and one referee complained of unspecific muscular symptoms in his right calf during the previous week). As a result, the study included 30 male referees.

The mean age of the participants was 22.24 years (SD 1.8, range 20–26 years). The anthropometric characteristics are presented in Table 1, while the summary results of the FIFA/AIA official tests and other field-based fitness tests are reported in Table 2.

The anthropometric measures of our study participants did not show significant correlations with the results of the FIFA/AIA official or other field-based fitness tests. The correlation coefficients between the results of FIFA/AIA official and other field-based fitness tests are reported in Table 3.

**Figure 2:**
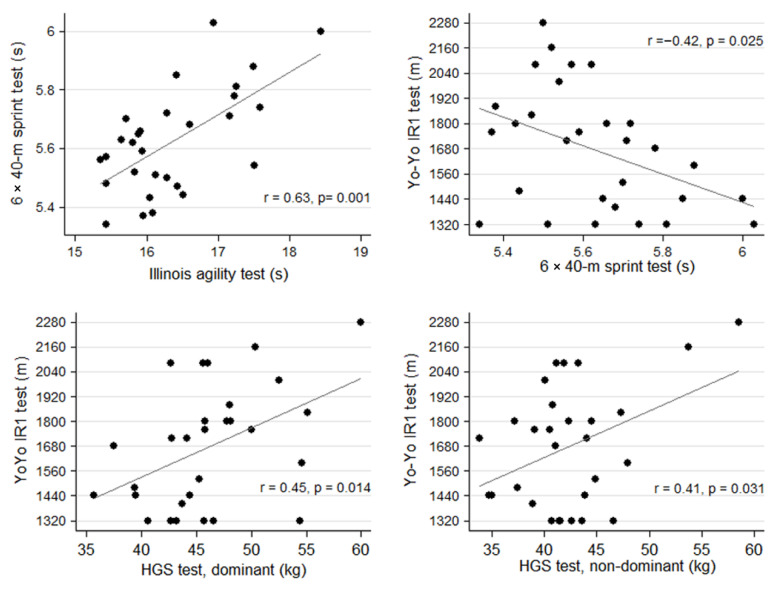
Scatter plots of the significant correlations between the results of FIFA/AIA official tests and other field-based fitness tests.

There was a strong positive correlation between the 6 × 40-m sprint and IA tests (r = 0.63, *p* = 0.001). A moderate positive correlation was observed between the Yo-Yo IR1 and HGS tests in the dominant (r = 0.45, *p* = 0.014) as well as non-dominant hand (r = 0.41, *p* = 0.031). Moreover, a moderate negative correlation was observed (r = −0.42, *p* = 0.025) between the two FIFA/AIA official tests, 6 × 40-m sprint and Yo-Yo IR1, indicating that the referees able to cover a longer distance in the Yo-Yo IR1 test were able to perform the 6 × 40-m test in a shorter time (Figure 2).

There was also a very strong correlation between the dominant and non-dominant sides in the HGS test (r = 0.72, *p* < 0.001). Finally, the results of the SLJ, SQJ, and SaR tests did not correlate with any of the FIFA/AIA official tests (r < 0.1).

## 4. Discussion

This study aimed to examine the correlation between two FIFA/AIA official tests used for the yearly evaluation of the fitness level of international and regional soccer referees and other field-based fitness tests, which could be potentially implemented to monitor referee fitness during the sportive season. We have demonstrated that the results of the 6 × 40-m sprint test correlated strongly with the results of the IA test, while the Yo-Yo IR1 test correlated with HGS in both the dominant and non-dominant hand. Interestingly, there was only a moderate correlation between the two FIFA/AIA-approved tests, namely the Yo-Yo IR1 and 6 × 40-m sprint tests.

FIFA recommends that two tests, 6 × 40-m sprint and high-intensity 150-m interval tests, be performed during official fitness screening of the soccer referees at least once a year. Two other FIFA-approved tests, dynamic Yo-Yo or Yo-Yo IR1, can be used optionally. The 6 × 40-m sprint test is relatively simple and evaluates the ability to perform repetitive sprints. The Yo-Yo IR1 is technically more complex and requires a specific learning phase from both referees and evaluators. Nevertheless, Yo-Yo IR1 is the most commonly used test for monitoring the ability to cope with intermittent exercise in team sports [9]. Performance in this test was related to high-intensity action during the match, maximum oxygen consumption (VO2 max), and heart rate response during high-intensity intermittent activity [16]. The same was not observed for the 150-m interval test [8]. Accordingly, AIA chose the 6 × 40-m sprint and Yo-Yo IR1 tests, whose results correlated highly with the referees’ performance during matches, as a method to evaluate the level of fitness of its referees and allow them to officiate in a specific category.

Due to the large number of activities scheduled for the referees during the sportive year, the official tests are usually performed at the beginning of the season. Their implementation during the year is generally infeasible. First, they require a specific place and time allocation; second, these tests impose a high physical demand on the referees. From the above observations, it follows that other field-based fitness tests need to be applied if the assessment of referee fitness was to take place during the sportive season, in between the scheduled activities.

The field-based fitness tests evaluated in the present study are commonly used under different physiological [14,17,18] and pathological conditions [19,20] and their validity has been extensively proved in other studies [10,21,22]. Together, they are able to assess several aspects of fitness status, and they are often performed in sequence, a so-called fitness battery, to cover a wide range of fitness domains. For the scope of our study, however, we chose to analyze the correlation between the FIFA/AIA official tests and the individual alternative tests, rather than all of them as a fitness battery. Mainly, this was to avoid increasing the physical strain of the task, which, in line with the hypothesis of the study, should be performed during the sportive season, in between scheduled officiating activities. The main advantage of these tests is their simplicity, as they require no or little equipment, they are not time-consuming (taking less than 5 min to complete), and they impose low physical demand.

The results of our study indicate that several common field-based fitness tests correlate significantly with the FIFA/AIA official tests. In particular, a strong positive correlation was present between the 6 × 40-m sprint test and the IA test. Although this correlation could seem obvious, as both tests are speed-related and are reported in seconds, they do not assess the same fitness domain. In the 6 × 40-m sprint, the referee has to sprint straight ahead, while the IA test requires a more complex pattern of running, with several changes in direction and combined phases of acceleration and deceleration. For these reasons, the 6 × 40-m sprint test is commonly considered a speed test, while the IA test is universally interpreted as an agility test. Undoubtedly, the characteristics of the IA test are highly pertinent to the actual activity performed by the referees during the match. Therefore, the observed high correlation between the results of these two tests is particularly relevant for testing our hypothesis.

Another correlation observed in the study was that between the Yo-Yo IR1 and HGS tests, for both the dominant and non-dominant side. The “two-sidedness” of this correlation can be in large part explained by a high correlation of HGS between both sides in the same subject, even though the scores for the dominant hand were usually significantly higher than for the non-dominant hand. The correlations were positive, which means that the referees able to cover a longer distance in the Yo-Yo IR1 test are able to reach higher scores in the HGS test, or vice versa. Apparently, this correlation is less intuitive. Indeed, the Yo-Yo IR1 test is an interval test covering resistance and lower limb strength—that is, musculoskeletal fitness and cardiovascular fitness—while the HGS test merely assesses the strength of the distal upper limb. Nevertheless, previous studies highlighted the correlation between the HGS test and exercise capacity, whether in healthy subjects [23], elderly people [24], or patients with pathological conditions [25,26]. Interestingly, the results of the HGS test correlated with the 6-min walking test and incremental shuttle walking test, which was developed to assess the functional capacity of patients with chronic airway obstruction. The latter one is indeed similar to the Yo-Yo IR1 test in structure. Singh et al. [27] observed a significant correlation between the HGS and incremental shuttle walking tests and concluded that the HGS test is the main determinant of patient performance.

Our study is not without limitations. The inclusion criteria were restricted to a specific regional category of referees with similar anthropometric characteristics. All participants were males aged 20–26 years, and they officiated in the same category. Thus, they were engaged in a similar athletic activity, consisting of training sessions and matches, throughout the sportive year. Although these characteristics guarantee the homogeneity of the cohort, they may limit the external validity and the generalization of the results. Although the sample size was limited, it was calculated to be statistically valid. As for the study protocol, all tests were performed during the same day and repeated in the same sequence on different days. Counterbalancing measures were not considered in the study design. The warm-up and the recovery phases between the tests were included to limit the impact of muscular fatigue on physical performance. Thus, in the authors’ view, a bias able to influence the results of the tests can be excluded.

## 5. Conclusions

In conclusion, the results of the present study show that a significant correlation exists between the 6 × 40-m sprint and IA tests and between the Yo-Yo IR1 and HGS tests. It is not our intention, however, to suggest that the IA and HGS tests should be substituted for the official FIFA/AIA tests. Instead, given the difference in the technical, physical, and physiological demands of these tests, they could be applied to the assessment of referee fitness in a complementary manner: 6 × 40-m sprint and Yo-Yo IR1 tests at the beginning of the sportive year for referee qualification and categorization, and IA and HGS tests during the sportive year for the systematic or even random evaluation of the referees’ fitness status during the whole season. At the same time, the latter two could be used in studies aimed at monitoring the impact of training sessions and officiated matches on referee fitness during the sportive year.

## Figures and Tables

**Table 1:** Anthropometric characteristics of the participants (*n* = 30).

Characteristic	Mean	SD	Range
Height (cm)	180.36	6.33	168–191
Weight (kg)	73.67	7.38	59.2–89
Waist circumference (cm)	78.35	5.97	67–93
BMI (kg/m^2^)	22.65	2.07	18.58–27.58

**Table 2:** Results of FIFA/AIA official tests and other field-based fitness tests (*n* = 30).

	Mean	SD	Range
FIFA/AIA official tests:			
6 × 40-m sprint (s)	5.63	0.18	5.34–6.03
YO-YO IR1 (m)	1678.62	292.67	1320–2280
Other field-based fitness tests:			
HGS, dominant hand (kg)	46.14	5.55	35.65–60
HGS, non-dominant hand (kg)	42.36	5.21	33.85–58.5
SLJ (cm)	225.88	17.52	201.5–265
SQJ (cm)	1093.03	85.59	926–1239
SaR (cm)	19.69	8.93	1.5–38
IA (s)	16.37	0.78	15.35–18.44

Table note: Yo-Yo IR1 = Yo-Yo Intermittent Recovery test, SLJ = standing long jump, SQJ = standing quintuple jump, SaR = sit-and-reach test, IA = Illinois Agility, HGS = hand-grip strength.

**Table 3:** Correlation coefficients between the results of FIFA/AIA official tests and other field-based fitness tests.

Test	6 × 40-m Sprint	YO-YO IR1
HGS, dominant	−0.30 (0.112)	0.45 (0.014) *
HGS, non-dominant	−0.18 (0.351)	0.41 (0.031) *
SLJ	0.04 (0.850)	0.02 (0.917)
SQJ	0.06 (0.772)	0.16 (0.401)
SaR	−0.01 (0.982)	0.28 (0.137)
IA	0.63 (0.001) *	−0.29 (0.119)

Table note: Data are reported as correlation coefficient, r (significance level, *p*). * denotes statistically significant correlation (*p* < 0.05).

## Data Availability

The datasets generated and analyzed during this study are available from the corresponding author upon reasonable request.
